# Ranking Malaria Risk Factors to Guide Malaria Control Efforts in African Highlands

**DOI:** 10.1371/journal.pone.0008022

**Published:** 2009-11-25

**Authors:** Natacha Protopopoff, Wim Van Bortel, Niko Speybroeck, Jean-Pierre Van Geertruyden, Dismas Baza, Umberto D'Alessandro, Marc Coosemans

**Affiliations:** 1 Department of Parasitology, Prince Leopold Institute of Tropical Medicine, Antwerp, Belgium; 2 Médecins Sans Frontières Belgium, Brussels, Belgium; 3 Department of Animal Health, Prince Leopold Institute of Tropical Medicine, Antwerp, Belgium; 4 School of Public Health, Université Catholique de Louvain, Brussels, Belgium; 5 Programme de Lutte contre les Maladies Transmissibles et Carentielles, Ministry of Health, Bujumbura, Burundi; 6 Department of Biomedical Sciences, Faculty of Pharmaceutical, Veterinary and Biomedical Sciences, University of Antwerp, Antwerp, Belgium; Sabin Vaccine Institute, United States of America

## Abstract

**Introduction:**

Malaria is re-emerging in most of the African highlands exposing the non immune population to deadly epidemics. A better understanding of the factors impacting transmission in the highlands is crucial to improve well targeted malaria control strategies.

**Methods and Findings:**

A conceptual model of potential malaria risk factors in the highlands was built based on the available literature. Furthermore, the relative importance of these factors on malaria can be estimated through “classification and regression trees”, an unexploited statistical method in the malaria field. This CART method was used to analyse the malaria risk factors in the Burundi highlands. The results showed that *Anopheles* density was the best predictor for high malaria prevalence. Then lower rainfall, no vector control, higher minimum temperature and houses near breeding sites were associated by order of importance to higher *Anopheles* density.

**Conclusions:**

In Burundi highlands monitoring *Anopheles* densities when rainfall is low may be able to predict epidemics. The conceptual model combined with the CART analysis is a decision support tool that could provide an important contribution toward the prevention and control of malaria by identifying major risk factors.

## Introduction

In recent decades, highland malaria has been a re-emerging problem in several African countries (Ethiopia, Uganda, Kenya, Tanzania, Rwanda, Burundi and Madagascar) [1], [2]. The spread of the vectors distribution in time and space exposes the human populations to a longer transmission season, resulting in a higher endemicity in the highlands [2], [3]. Besides, deadly epidemics have been reported with higher frequency and amplitude than before [4]–[8]. Indeed, one fifth of the African population lives in malaria epidemic prone areas (desert fringes and highlands) [9] where all age groups are at risk of clinical malaria due to the limited acquired immunity. The prevention of malaria in these vulnerable populations is one of the priorities for African leaders and international agencies [10]. It is therefore, essential to understand the factors fuelling these changes in transmission so that a national strategy plan for epidemic prevention and control can be developed in highland regions.

Former reviews published in 1998, have already shown the complexity of factors influencing malaria in the highlands [1], [2]. The aim of the present paper is to summarise and update current knowledge on malaria in the African highlands and build a detailed conceptual model for malaria risk factors. Furthermore, the hierarchical importance of these factors in influencing highland malaria is analysed using classification and regression trees [11], [12] (CART). The CART method is useful when dealing with large numbers of explanatory variables and to explore the relationship and the relative importance of these variables as well as all their possible interactions [13]. Therefore, the conceptual model associated with a CART analysis may be used as a decision support tool and different strategies could be implemented according to the risk factors that emerge as the strongest.

The CART method has been applied to the case of Burundi [14], [15] and measures to control and/or prevent malaria epidemics are discussed.

## Methods

### Conceptual Model of Malaria Risks Based on Literature Review

Based on a literature review, different risk factors for malaria in African highlands were identified and used to build a conceptual model. The main source of information was peer-reviewed scientific papers obtained through PubMed using the keywords “malaria” and “highland”. Both English and French papers, describing malaria potential risk factors, were used. The reported risk factors were classified according to their impact on vectors or on malaria. To determine the hierarchical importance of different risk factors identified in the conceptual model the Classification and Regression Trees (CART) were used on malaria data collected in the Burundi highlands.

### The Burundi Database

A four year vector control programme based on one annual round of Indoor Residual Spraying (IRS) was carried out between 2002 and 2005 in the central highland province of Karuzi, and targeted the valleys where malaria transmission was the highest [14]. Long Lasting Insecticidal Nets were also distributed in 2002. Between 2002 to 2007, bi-annual (May and in November) cross sectional surveys (11 surveys in total) were carried out. The sampling process has been described in detail elsewhere [14], [15]. Briefly, during each survey 450 to 800 houses were sampled and in each of them (total houses sampled for the 11 surveys  = 8075), *Anopheles* were collected with the spray catch method and a blood slide of two randomly selected persons (≤9 and >9 years old) were taken (total person included in the 11 surveys  = 12745, 36% of the houses have no children ≤9 and in 6% of the houses one of the selected person was not present). Of the 14,932 *An.gambiae* and *An.funestus* that were collected, 244 were found positive for the detection by ELISA of the *P.falciparum* circumsporozoite antigen (Wirtz). The intervention was evaluated on the basis of the reduction of the *Anopheles* density, infective bites by house by month and the prevalence of malaria infection. Information on location, housing construction (house size, open eaves, type of wall and roof), livestock, separate kitchen, vector control activities (net use and spraying), prior antimalarial treatment, sex and age was also collected. Altitude and distance to the marsh of the houses were registered with a hand held positioning system (GPS 76, Garmin®). Average monthly minimum and maximum temperatures and monthly rainfall, recorded at the Karuzi meteorological station, were obtained from the Institute of Geography of Burundi (IGEBU).

A verbal informed consent was obtained for the blood slides and mosquito collections. For children the consent was obtained from the parents. In case of refusal, other persons or the next household was asked for consent. Present procedure and the full study was approved by the Commission of Medical Ethics of the Prince Leopold Institute of Tropical Medicine Antwerp (Belgium) (ref number 04 26 4 461). At the time of the implementation of the study, the Institutional Ethical Committee was not functional in Burundi. However, the Ministry of Health signed an agreement for the vector control program and the study design and the national malaria control program (LMTC) offered close collaboration.

### The Classification and Regression Trees CART

The non-parametric classification and regression tree (CART) models were used to explore the influence of the specified determinants on the level of malaria and on the *Anopheles* mosquitoes. CART models are useful tools to explore the interactions between a desired outcome and its determinants [13], [16]. They can be used to analyse either categorical (classification) or continuous data (regression). The analysis was performed using a commercial software CART (Salford systems Inc. Version 6, California, USA).

CART expresses its result in the form of a decision tree, a different approach that the better known parametric techniques. Indeed, in classical regressions the linear combinations are the primary method of expressing the relationships between variables while in CART this does not need to be linear or additive and the possible interactions do not need to be pre-specified or of a particular multiplicative form. The decision tree resulting of CART is useful, as well as the resulting flexibility and the non-parametric form (no assumption upon the covariates). The CART has many advantages, but they are known as instable approach. Therefore in the present paper we used a 10-fold cross-validation as estimation method.

The building of a classification tree begins with a root (parent) node, containing the entire set of observations, and then through a process of yes/no questions, generates descendant nodes. Beginning with the first node, CART finds the best possible variable to split the node into two child nodes. In order to identify the best splitting variable (called splitters), the software checks all possible variables, as well as all possible values of the variable to be used to split the node. In choosing the best splitter, the program seeks to maximize the average “purity” of the two child nodes. The splitting is repeated along the child nodes until a terminal node is reached. Each terminal node is characterized by an average and a standard error (computed as the standard deviation divided by the square root of the terminal node size), indicating the purity of the node. The node purity measure provides an indication of the relative homogeneity (the inverse of impurity) of cases in the terminal nodes. If all cases in each terminal node show identical values, then node impurity is minimal, homogeneity is maximal, and prediction is perfect (at least for the cases used in the computations). In this study, the Gini criterium and the interclass variance were used as a measure of “purity”.

The one standard error rule was applied to select the best tree (the smallest tree within 1 standard error of the minimum error tree). A minimum terminal node size of 500 samples was selected to avoid too many splits, with few observations, that are difficult to explain.

CART also provides a ranking based on the overall contribution of each variable in the construction of the tree. This ranking indicates the importance of each independent variable as a predictor. Importance, for a particular variable, is the sum across all nodes in the tree of the improvement scores between this variable and the best splitter at a particular node [17]. It is thus possible that a variable enters the tree as a second most important splitter in many nodes (and will not appear on the tree), but never as the primary splitter. However, such variable will turn out as very important in the overall variable ranking. The advantage of such an approach is that important contributing determinants are not ignored.

### Multivariate Analysis

The risk of a positive slide was analyzed using the survey logistic regression in Stata 9.2 (Stata Corporation, College station, Texas, USA), taking into account the study design. Negative binomial regressions were used for the analysis of *Anopheles* density.

## Results

### Conceptual Model for Malaria Risk in the Highlands

**Figure 1:**
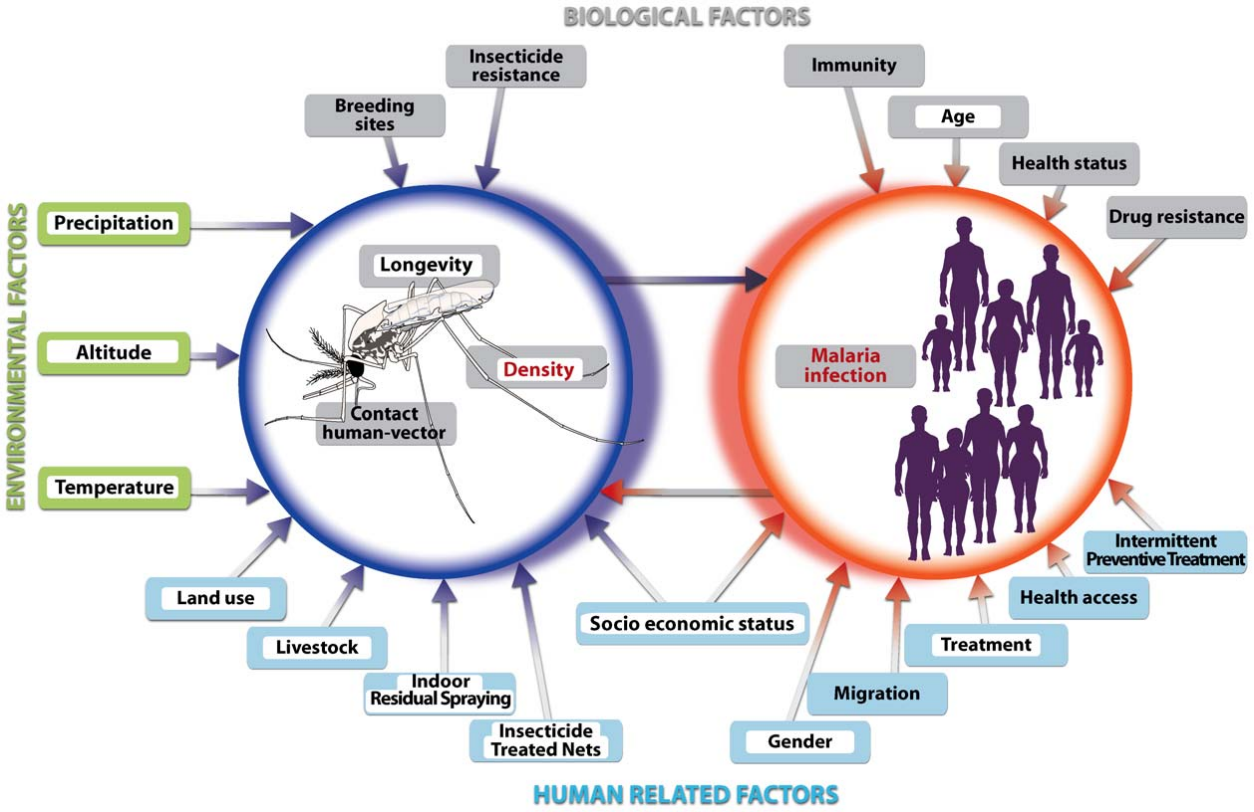
Conceptual model of important risk factors affecting malaria prevalence in the African Highlands. Factors are regrouped in 3 main classes (environmental factors: green label, biological factors: grey label and human related factors: blue label). Dependant variables included in the CART analysis are displayed in red and predictor variables are highlighted in white.

Due to the instability of transmission in the highlands small variations in environmental or human related factors can have dramatic consequences for malaria transmission due to the low immune status of the human population [2], [18]. Different factors can drive these changes by influencing the vector's transmission capacity and the malaria prevalence. These factors can be grouped into three classes: (1) environmental factors such as altitude and climate (2) biological factors related to the *Anopheles* vector, the parasite and the human host and (3) human related factors such as socio-economic status, health access, migration, gender, control activities (IRS, Insecticide Treated Net, and Intermittent Preventive Treatment) and land use (irrigation, deforestation, swamp drainage and living near breeding sites). The conceptual model of potential factors influencing either *Anopheles* (density, longevity or/and contact with human) or the outcome of transmission (i.e. malaria infection) in the highland based on this review is presented in Figure 1.

### Factors Influencing Malaria

The ability to suppress malaria infection depends on immunity. It has been suggested by Bodker et *al* [18] that acquired immunity is both exposure and age-dependent. At a moderate level of transmission (0.1 to 2 infective bites per year), immunity will develop with increasing transmission but after a certain age (2–3 years) the immunity will increase independently of transmission intensity. In low transmission areas, however, prevalence of infection and clinical malaria is similar in all age group.

The health status of the population can have an important impact on malaria infection. Malnutrition can weaken children's immunity and can increase the level of malaria morbidity and mortality [19], [20]. HIV has been associated with an increased level of malaria transmission in South Africa [21] and might enhance malaria parasite biomass [22]. It has also been observed that in all endemic areas the frequency of malaria infection is greater in pregnant women than in non pregnant women. However in low or unstable transmission areas, as in the African highlands, the effect of parity is less pronounced or even absent as compared to high transmission areas [23], [24]. Conversely, the implementation of intermittent preventive treatment is able to reduce morbidity in pregnant women [25] and in infants [26].

In several countries, the resurgence of malaria has been largely attributed to the emergence and spread of drug-resistant parasites [27]–[31]. The progressive build-up of the gametocyte pool in the human reservoir, contributing to the speed-up of transmission, could be enhanced by treatment failure of sulphadoxine-pyrimethamine [32] and chloroquine [33]. In contrast, implementation of effective treatment, such as artemisin-based combination therapy, has improved cure rates, decreased the gametocyte carriage and, therefore, resulted in a reduced transmission in low endemic areas [34], [35].

Finally, other human related factors, such as population migrations [36], reduced health systems access and quality [2], and socio-economic pressure as population growth [37], [38] have also created favourable conditions for malaria outbreaks.

### Factors Influencing the Vector

Mosquitos' longevity, man-vector contact and mosquito density determine the transmission capacity of a vector population. First, a reduction in the lifespan of the mosquitoes will reduce the sporozoite rate and hence the proportion of infective bites. Secondly, a reduction in the human/vector contact will decrease the proportion of blood meals taken on human hosts. Finally, a reduction of vector density by decreasing the number of adult or larvae will also reduce transmission intensity. Therefore, any factors that could have an impact on any of these components will influence malaria transmission. According to the MacDonald model [39], factors influencing longevity will have more impact on transmission than factors affecting human-vector contact or density.

An altitude around 1800–2000 meters is usually considered the upper limit at which malaria transmission occurs [2], though epidemics have been recorded higher [19], [40]. The protective effect of altitude is linked to the decreasing temperature (0.5°C to 0.7°C every 100 meters) [41], [42] that increase the length of the extrinsic incubation period and hence decrease the likelihood of a mosquito of becoming infectious. Optimum conditions for the extrinsic development of the parasite are between 25°C and 30°C [41]. Below 16–19°C, few vectors survive before the completion of the sporogonic cycle and this temperature range is often considered as the threshold for stable malaria transmission. Temperature will also influence the longevity and feeding frequency of a mosquito [41]. The aquatic stage of anopheline is also temperature-dependant. In the laboratory, it was observed that larval mortality increased considerably when water temperature fell below 18°C [43]. In the Kenyan highlands only a small larval survival rate was observed at low temperatures [44], while the adult mosquitoes could survive inside houses with temperatures 2 to 3 degrees higher [40], [45]. Therefore, a small temperature rise either through seasonal variability [46], local microclimatic changes due to modification in vegetation cover [47]–[49] or to global warming [50]–[52] can increase malaria transmission and distribution.

Rainfalls play a crucial role in malaria epidemiology by providing breeding sites for the aquatic stages of the mosquito's life cycle. In addition, rainfalls may increase the relative humidity; above 60%, adult mosquitoes longevity, and consequently the vectorial capacity, increases [41]. By contrast, heavy rain showers can flush away and kill larvae [53]. In Ethiopia [19] and Uganda [5], [54], for example, extreme rainfalls were associated with malaria epidemics, whereas in Tanzania malaria decreased after intense rains [55].

In highland settings, malaria transmission is negatively correlated with the increasing breeding sites-house distance [14], [56], [57]. The topography of highland areas affects the spatial distribution of breeding sites [58] and land use changes such as irrigation [59]–[61] and swamp drainage for cultivation [49], [62], [63], or for other economic activities [64] can create new habitats for malaria vectors such as *Anopheles gambiae* and *Anopheles funestus*. Environmental management, on the other hand, can reduce the availability of the breeding sites and therefore decrease the vectors density population [65]–[67]. Vector control measure such as Indoor Residual Spraying (IRS) and Insecticide Treated Net (ITN) have a high impact on transmission by reducing both *Anopheles* survival and density, if the coverage is high [14], [68]. ITN can also decrease the man-vector contact and reduced the human blood feeding success [69], [70]. However, the emergence of insecticide resistance may decrease the effectiveness of these methods [71], [72].

Household and socio-economic factors have also an impact on malaria transmission by influencing the human-vector contact. For example, more mosquitoes were found in poorly constructed houses as compared to good ones [73], [74]. Other factors such as keeping livestock inside the house, type of roof, open eaves, no separate kitchen were also associated with increase malaria risk [74], [75].

### Identifying and Ranking Malaria Risk Factors in Burundi Highlands

Based on the conceptual model (Figure 1), variables among the three classes (environmental, biological and human related) were chosen from the Burundi database and used in the CART analysis to determine their impact either on the malaria infection or on *Anopheles* prevalence. Predictive factors for malaria infection included in the CART analysis were *Anopheles* density, density of infected *Anopheles*, individual human characteristics (age and sex), housing condition, past treatment, and “survey” (Table 1). The variable “survey” was included in the prevalence model and represents the possible time-related variables not covered by the parameters included in the vector density and prevalence analyses. To study the impact on *Anopheles*, two regression trees were developed, one with as a dependant variable the *Anopheles* density and the other with as a dependant variable the density *Anopheles* infected with *Plasmodium falciparum*. The factors included were monthly rainfall and average monthly minimum and maximum temperatures (during the month of the survey and 1–2 months before the survey), housing conditions, vector control activities, and environmental parameters. Detailed information of the houses was available on the Burundi database. The characteristics of these houses were scored by size and type of construction: house size (floor area size score 1 =  <25 m^2^, score 2 = 25–50 m^2^, score 3 =  >50 m^2^), type of wall (1 =  thatch, 2 =  mud, and 3 =  bricks), type of roof (1 =  thatch, 2 =  iron sheet, and 3 =  tiles) and separate kitchen (1 =  no, 2 =  yes). The score of the houses' characteristics were combined and divided into four categories (score 4–5 = **1**: poorest housing condition, score 6–7 = **2**, score 8–9 = **3**, score 10–11 = **4**: best housing condition). Other factors presented in the conceptual model were not collected in our study and could not be included in the analysis.

**Table 1:** Dependant and predictor variables introduced in the CART analysis.

Dependant Variable	Variables classes	Predictor Variables
***Anopheles*** ** density**	**Environmental**	
	○ Precipitation	Current monthly rainfall (mm)
		Lagged monthly rainfall: one month (mm)
		Lagged monthly rainfall: two months (mm)
	○ Temperature	Lagged average monthly minimum T°: one month (°C)
		Lagged average monthly minimum T°: two months (°C)
		Lagged average monthly maximum T°: one month (°C)
		Lagged average monthly maximum T°: two months (°C)
	○ Altitude	Altitude houses (m)
		• **≤**1450
		• 1451–1500
		• 1501–1550
		• 1551–1600
		• 1601–1650
		• >1650
	**Human**	
	○ Land use	Distance to marsh (m)
		• ≤300
		• 301–500
		• 501–700
		• 701–900
		• 901–1100
		• >1100
		Type of crop in the marsh
		• Two crops/year: rice and vegetable
		• Rice field
		• Vegetable
		• Few crop
	○ Housing	Houses: Poor constructions to better (4 categories)
	○ Livestock	Keep livestock in the houses (yes/no)
	○ IRS	Houses in sprayed areas (yes/no)
	○ ITN	Use of insecticide treated nets (yes/no)
**Positive slide**	**Biological**	
		Age (year)
		Sex
		*Anopheles* density (number/houses)
		Density of infected *Anopheles* (number/houses)
	**Human**	
		Past treatment during the 3 previous months (yes/no)
		Keep livestock in the houses (yes/no)
		Sleep under a net (yes/no)
		Houses: Poor constructions to better (4 categories)
	**Other**	
		Survey

**Figure 2:**
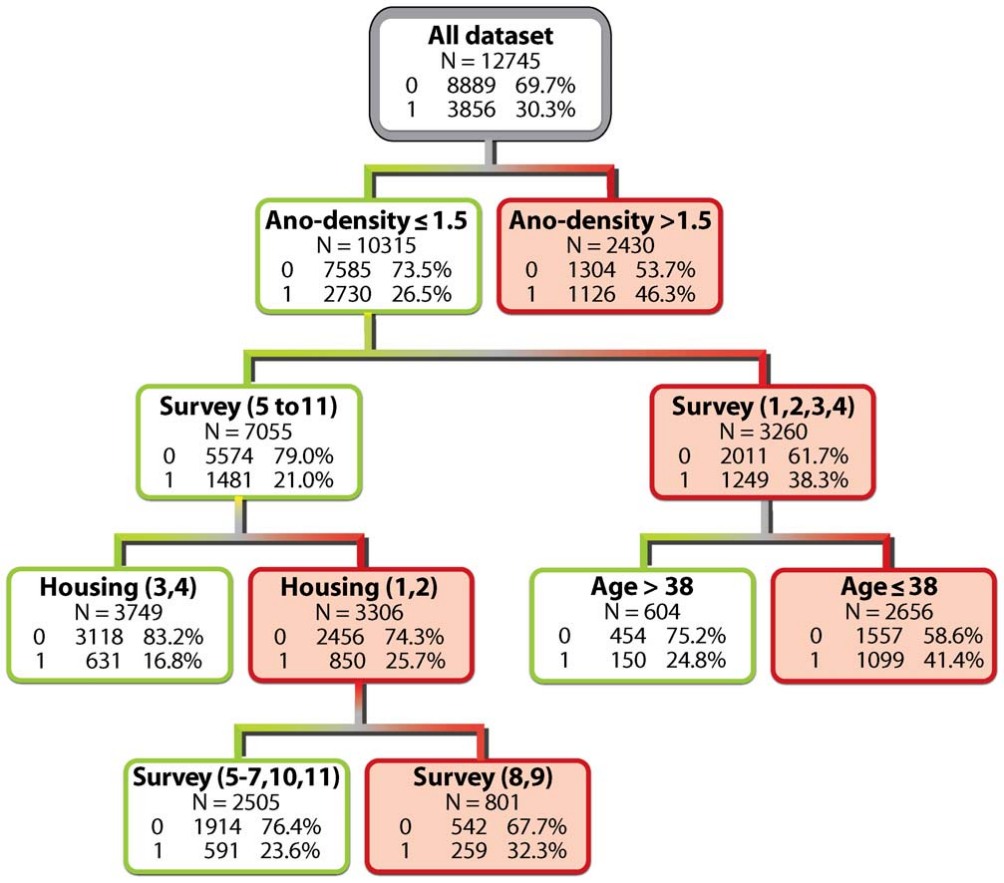
Classification trees representing the important risk factors for malaria prevalence. The high risk groups are displayed in red. In each node 0 stands for negative slide and 1 for positive slide. The following variables were selected by the tree as important risk factors: *Anopheles* density (Ano-density) with a cut off of 1.5 *Anopheles* per house; Survey number 1 to 11; Housing (1,2 =  poorest housing condition and 3,4 highest housing condition); Age with a cut off of 38 years old.

The overall ranking of predictor variables for malaria infection is presented in Table 2. Surveys and *Anopheles* density were the two most important factors. Housing conditions that influence the human-vector contact and infective mosquitoes were ranked third and sixth. In the corresponding classification tree, *Anopheles* density was the first splitter (Figure 2) with a higher prevalence (46.3%) in houses with more than 1.5 *Anopheles* compared to houses with fewer *Anopheles* (prevalence: 26.5%). Then, in this last group, malaria prevalence was lower in surveys 5 to 11 (year 2004 to 2007) compared to surveys 1 to 4 (year 2002 to 2003). For surveys 1 to 4, age was the best discriminator with a threshold at 38 years; for survey 5 to 11, malaria prevalence among people living in the poorest houses was higher (25.7%) than for people living in the better-constructed ones (16.8%). Each terminal node is categorised as 1 (positive) or 0 (negative) depending on whether the proportion of 1's exceeds the proportion of 1's in the population (30.3%). From all negative individuals, 62% (5486/8889) were properly classified as negative (0) and from all positive individuals, 64% (2484/3856) were properly classified as positive.

**Table 2:** Ranking of predictor variables for malaria prevalence by their overall power as discriminant.

Variables	Power
Survey	100
*Anopheles* density	91.0
Housing	23.4
Age	22.8
Past malaria treatment	5.8
Density of infected *Anopheles*	1.0
Livestock in houses	0.9
Sleep under a net	0.0
Sex	0.0

**Figure 3:**
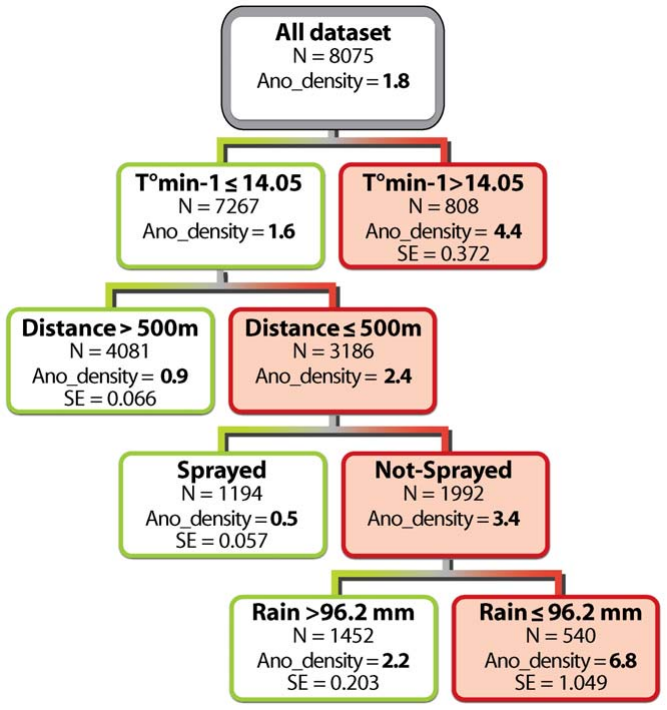
Regression trees representing the important risk factors for the *Anopheles* density per/house (Ano_density). The selected splitting variables (Minimum temperature the previous month  =  T°min-1; Distance of the houses to the marsh with a cut off of 500 metres; Area sprayed or not; Monthly rainfall in the current month with a cut off of 96.2 mm) are shown in the nodes.

As the *Anopheles* density was more important than density of infected *Anopheles* for the malaria prevalence (Table 3), only the regression trees with the dependent variable *Anopheles* density is presented. According to their overall discriminatory power, monthly rainfalls in the current month and with one month time lag, emerged as the two strongest predictors for *Anopheles* density, followed in decreasing order of importance by spraying, net-use, monthly minimum temperature with one and two months time lag, distance to the marsh, altitude of houses, and two months lagged rainfall (Table 3). The resulting regression tree is presented in Figure 3. Every important predictor divides a node into two sub-nodes, either with a higher or a lower *Anopheles* density. A one month lag minimum temperature was the main splitter, with temperature below 14.05°C being associated with the lowest *Anopheles* density (mean: 1.6/house). This sub-node was further split by the variable “distance to the marshes”. The highest *Anopheles* density (2.4/house) was found in houses located within 500 metres from the marsh. Spraying was then the best discriminative variable for this node, *Anopheles* density being the highest (3.4/house) when houses were not in treated areas. In this group, current rainfall had an influence over the average *Anopheles* density. Monthly rainfalls higher than 96.2 mm were associated with lower *Anopheles* density (2.2/house). Monthly rainfalls in the preceding month (power: 99.9), net use (power: 83.1) and a lag minimum temperature of two months (power: 73.0) did not appeared as main splitters in the final tree whereas they were identified as important risk factors for *Anopheles* density as shown by the ranking of their discriminatory power (Table 3). This happens because at many stages in the tree building they are important but never as important as the main splitter.

**Table 3:** Ranking of predictor variables for *Anopheles* density by their overall power as discriminant.

Variables	Power
Rain	100
Rain - 1 month	99.9
Spraying	96.7
ITN use	83.1
T°min - 1 month	75.7
T°min - 2 months	73.0
Distance from the marsh	42.3
Altitude of the houses	31.9
Rain - 2 months	22.4
Type of crop	9.3
T°max - 1 month	5.7
T°max - 2 months	5.1
Housing	0.0
Livestock in houses	0.0

The logistic regression, showed the surveys, density of *Anopheles*, type of house, age and density of infected *Anopheles* to be significant (results not shown). In the negative binomial regression the following variables were retained in the model: rain with one and two months time lag, spraying, net used, minimum temperature the previous month, distance to the marsh, altitude and presence of animals in the house.

## Discussion

The conceptual model used in this study provides insight into a complex disease, malaria, by listing all the potential risk factors influencing the transmission capacity of the vectors and the malaria prevalence in the human population. Such complexity requires tools that enable to explore the hierarchical importance of these risk factors. CART is such a tool and has proven its adequacy and usefulness in other contexts, for example for bovine spongiform encephalopathy [76]. In the Malaria field, CART methods (or similar approaches) were used for spatial analysis of malaria risk [77] and has been also used recently to report the accurate and dynamic picture of the main risk factors for malaria infection in Vietnam [12].CART has also the advantage to be user and reader friendly, generating results comprehensible for a wider audience. Therefore, the conceptual model-CART approach will lead to a better understanding of the local malaria epidemiology and a better targeting of control efforts.

Variables of high importance in both CART and parametric analysis are almost the same, except for rain which is non significant in the logistic regression. This can probably be explained by high colinearity with other rain variables (with time lag of one and two months). A CART Analysis works with (non-predefined) interactions. This means that the only similarity between a parametric model and a CART model will be the first split. The variable “animals” was ranked last in CART, probably because it is important only at the first step in the CART tree (in analogy with a parametric model) but will only contribute a small part to the final importance which is obtained by adding contributions at different splits in the tree.

In the Burundi highlands, *Anopheles* density, hence vector density because 95% of the collected *Anopheles* mosquitoes in the study area were malaria vectors [14], was the second best predictor for malaria infection in the human population. The infective mosquitoes were only the sixth most important predictor variable in the overall ranking. Likewise, Bodker *et al* [78] found that a decline in malaria transmission due to altitude is primarily due to a diminution of vector abundance and, to a lesser extent, by a reduction in the proportion of infective mosquitoes. In other studies, such as in Kenya and Madagascar, the importance of vector density in unstable highland malaria has been highlighted [60]. Indeed, in these countries the introduction of irrigated rice fields increased the number of breeding sites available and exposed the non-immune population to higher transmission. Nevertheless, in endemic areas this is not always the case, as sometimes very high vector densities may result in a low vectorial capacity [79].

In the current study, important variations in prevalence between surveys were observed and cannot be explained by any of the factors included in this analysis. Some variables such as health access, health status, and migration were not collected during the surveys. Differences between the first four surveys (year 2002 and 2003) and the later ones could be attributed to the normal decline of malaria prevalence after the malaria epidemic of 2001 [80], or/and to the introduction of the artemisin-based combination therapy and Rapid diagnostic tests in December 2003. Mosquitoes and blood samples were collected at the same time. However, when considering the time needed for the parasite to develop in the vector and the human host, postponing the parasitological surveys by about one month could have improved the predicting power of *Anopheles* density on malaria prevalence. It is surprising that ITN-use was not associated with lower malaria prevalence while it affects the *Anopheles* density, a good predictor of malaria infection. This finding can be explained by the absence of any additional impact of ITNs on transmission once an almost full coverage of IRS has been achieved [14].

In the highlands of Burundi, the CART analysis shows that factors responsible for high *Anopheles* density are in agreement with other studies, i.e. lower rainfall [55]–[57], no vector control implementation in houses [75], higher minimum temperatures [46], [81], and breeding sites proximity [56], [57]. In Karuzi, high rainfalls, current or during the previous month, have a negative effect on vector densities. Minimum temperatures with 1 or 2 months time-lag are good predictors of vector density, while housing conditions and livestock in houses have no predictive value. This is most likely due to the predominance of the very anthropophilic vector *An. gambiae* s.s. (Form S) [82] in the study area (98.2% of the complex) [14]. In the presence of *An. arabiensis*, however, it has been observed that keeping cattle in houses was a risk factor for malaria [74], [83]. It is also obvious that vector control (ITN or IRS) using pyrethroids insecticides reduces vector density in treated houses due to either the excito-repellent effect of this class of insecticides or by their mass killing effect. Monthly cumulated temperature and rainfall data have been used in this work to assess the importance of environmental factors. However to improve the predictive value, weekly data in different locations would me more appropriate especially in the highland where environmental factors can vary greatly between valleys and where precipitations are non homogenous through the month.

Climate (temperature and rainfall) is an important determinant of malaria vectors in the highlands. An increase in temperature and a modification in the frequency or amount of rainfall would affect future transmission of malaria. Different biological and statistical models have assessed the potential geographical expansion of malaria ranging from small change in the next decades [84], [85] to substantial extension by the end of the century [86], [87]. However, the models mostly focused on the effect of temperature on parasites development and vectors' longevity and should now evaluate the impact of climate warming on *Anopheles* density. The attribution of malaria resurgence observed in recent decades in African highlands to climate change is, however, controversial. Whereas some studies have associated warming trends to the increase in malaria transmission [50], [52], [88], other studies show no association [8], [89]–[91]. In Burundi, Bonora *et al* [92] attribute the upsurge of malaria infection in the highlands, leading to the 2000 epidemic, to climate warming. However, in Karuzi, temperatures were recorded since 1988 and no warming trend in monthly mean, maximum and minimum temperature was observed until the 2000 epidemic [93]. An unusually high precipitation occurred at the time of the malaria peak (November) and could not be responsible for triggering the epidemic, but was probably responsible for stopping or decreasing malaria transmission [53]. For instance, it is commonly known that high rainfalls trigger malaria epidemic whereas in our analysis lower precipitations were associated with high *Anopheles* density, a powerful predictor of malaria infection. The long dry season preceding the epidemic of 2000 [80] may thus partially explain the outbreak.

Monitoring the most important malaria risk factors will help to more adequately prevent and control increases in malaria. The forecasting or early detection of meteorological variability could give time for the implementation of control measures. For example, in the Burundi, a more careful monitoring of the impact of rainfall and temperature variability on *Anopheles* density should be further evaluated and a threshold risk set up in different areas. If such rainfall variability could be assessed a few weeks in advance, the follow up of the residual *Anopheles* densities when rainfall is low could help in predicting or early detecting epidemics [94]. However, the practical use of these data for early warning and especially the initiation of expansive control interventions based on it, need to be assessed especially in the highlands [95].

It is commonly known that decrease in mosquito longevity will be more effective to limit transmission than reducing mosquito density [39]. Therefore, environmental management aiming at larval source reduction will contribute little to the overall decrease of malaria burden [96]. However, this activity may be re-considered when *Anopheles* density is the most important factor determining malaria infections; any means aiming at reducing the *Anopheles* population will have an impact on malaria. In the past, successful malaria prevention programmes targeting larval habitats were implemented [97], [98] even in Africa [66]. During the last few years renewed consideration has been given to environmental management [99] with a recent successful implementation reported in the highlands of Ethiopia [67]. To be fully effective, such a method necessitates substantial information on vector ecology, distribution of breeding sites and local environmental conditions and should be combined with insecticidal adult vector control [100]. Indeed, in our model, IRS and ITN are strongly related to *Anopheles* density.

In conclusion, the conceptual model of highland risk factors in combination with a CART analysis can be considered as a simple decision support tool to better understand malaria epidemiology in various high altitude settings. The ranking of risk factors will help to prioritize monitoring, prevention and control efforts to the most important identified factors.
